# Preliminary Assessment of the Effect of a Flipped Classroom Combined with Mind Mapping on Learning Outcomes in Ultrasound Use in Animal Husbandry Education: A Comparison with Traditional Lecture-Based Learning Among Third-Year BS Students in China

**DOI:** 10.3390/ani16142129

**Published:** 2026-07-09

**Authors:** Xiangqi Hao, Haigang Wu, Zhehui Qu, Guangqiang Zhang, Kaiwei Deng

**Affiliations:** 1 Department of Veterinary Medicine (Veterinary Imaging and Diagnostic Ultrasound), College of Animal Science and Veterinary Medicine, Xinyang Agriculture and Forestry University, Xinyang 464000, China; 2 Engineering and Technology Research Center for Waterfowl Resources Development and Utilization and Epidemic Disease Prevention and Control of Henan Province, Xinyang 464000, China

**Keywords:** education, veterinary, flipped classroom, mind mapping, veterinary ultrasound, animal husbandry

## Abstract

To increase teaching effectiveness in the context of veterinary ultrasonography, we employed a flipped classroom combined with a mind mapping methodology for ultrasound use in animal husbandry education. A total of 61 students (experimental class, n = 30; control class, n = 31) participated in this study. Students in the experimental class prepared and presented group topics over a two-week pre-class period and subsequently engaged in question-and-answer sessions. Next, mind maps were generated with the assistance of AI tools, thus establishing a systematic knowledge framework. An in-class test (the Mann–Whitney U test) was administered after the lesson, and a 6-item questionnaire was designed to evaluate students’ levels of satisfaction. The combination of a flipped classroom with mind mapping was associated with a rightward shift in the score distribution, with more students in the experimental group scoring 90–100 (7 vs. 2). The Mann–Whitney U test revealed no statistically significant differences between the groups (U = 361.0, *p* = 0.135, r = 0.22), although the experimental group had a higher mean score (83.53 vs. 79.74). Most students endorsed the teaching model. The observed trends and positive student feedback from this preliminary assessment suggested that this approach may be a promising method for veterinary ultrasound education.

## Simple Summary

Veterinary ultrasonography is a core course in veterinary clinical medicine. This course involves complex theoretical concepts and is highly specialized, thus making it rather difficult to grasp. Traditional teaching methods tend to be teacher-centered, resulting in low levels of student engagement, limited classroom interaction, and generally poor teaching efficiency. Therefore, the selection of appropriate and effective teaching methods is crucial for enhancing the clinical reasoning skills of veterinary students. The present study employed flipped classrooms and mind maps to enhance students’ active engagement and help them understand the relevant conceptual framework. Overall, in this cohort, the combination of a flipped classroom with mind mapping tends toward higher mean quiz scores and more top-scoring students, in addition to positive student feedback.

## 1. Introduction

Veterinary imaging is a specialist module in veterinary clinical medicine that focuses primarily on the ultrasound characteristics of various animal tissues and organs in both healthy and diseased states. It aims to equip students with the theoretical knowledge and practical skills that they need for veterinary ultrasonography while simultaneously enhancing their ability to observe, analyze and diagnose the ultrasound features associated with common diseases [1]. However, in our teaching practice, we observed that students in the veterinary ultrasound class predominantly adopt a passive learning approach and have limited clinical reasoning skills and ability to interpret ultrasound findings. This course involves complex theoretical concepts and is highly specialized, thus making it rather difficult to grasp. Traditional teaching methods tend to be teacher-centered and rely on a rote ‘cramming’ approach to imparting knowledge, with limited classroom interaction. Consequently, students exhibit low levels of engagement, and their teaching efficiency is generally poor. Contemporary students, who have grown up in a digital environment, prefer interactive and self-directed learning over passive lectures [2]. Therefore, the selection of appropriate and effective teaching methods to enhance the clinical reasoning skills of veterinary students is crucial. In nursing education, research has indicated that flipped classrooms and mind mapping can not only enhance students’ active engagement but also help them grasp the relevant conceptual framework [3].

In traditional teaching, the teaching method is predominantly teacher-centered, with students understanding, absorbing and synthesizing knowledge through classroom lectures and postclass tests [4]. However, in the flipped classroom, teachers transition from being knowledge transmitters to learning facilitators, whereas students transform from being passive knowledge recipients to being active researchers. The teaching format also switches from classroom lectures and postclass tests to preclass learning and in-class inquiry and discussion. In the flipped classroom, students can engage in independent learning through various methods, such as by watching instructional videos, using artificial intelligence (AI) tools to search for learning materials, and interacting by learning platforms, as has been reported in the context of dental education [5]. During lessons, students deliver group PowerPoint presentations, share their reflections, and engage in collaborative discussions among groups. At present, the flipped classroom is being applied across a wide range of academic disciplines, demonstrating particular strengths in higher education, particularly in medical education [6]. In the context of veterinary medicine teaching, the flipped-classroom methodology has been implemented in some courses. This teaching framework enables tutors to monitor students’ learning progress in a timely manner while positively influencing the learning process. Ultimately, it offers new approaches and methods for enhancing the quality of undergraduate teaching and promoting educational reform [7,8,9].

Mind mapping has been adopted by educational institutions in many countries as a teaching tool, thereby driving the development of teaching across various disciplines. In China, mind mapping has also begun to be incorporated into the teaching of numerous subjects and has rapidly become a focal point of research on educational reform [3]. Furthermore, mind mapping has achieved remarkably positive results with respect to the reform of courses such as pediatric nursing education [10] and medical laboratory education, including clinical molecular biology testing technology [11] and medical diagnostics [12]. With the increasing popularity of AI tools, a few studies in the fields of healthcare and nursing education have used these tools to generate mind mapping for teaching and have achieved positive results [13].

The application of ultrasound in animal husbandry is a compulsory module within the veterinary ultrasonography course, with a strong emphasis on practical application and hands-on experience with pigs, cattle and sheep, thereby providing vital information for students who intend to pursue a career in veterinary clinical practice. In our study, we employed a combination of a flipped classroom with mind mapping to shape the teaching of ultrasound applications involving pigs, cattle and sheep. The flipped classroom was designed to cultivate students’ independent learning skills and innovative thinking, while the knowledge frameworks constructed on the basis of mind maps facilitated a reduction in the difficulty of memorizing specialized knowledge and improved learning efficiency. Mind mapping supported the flipped classroom by helping students engage in independent learning, thus allowing them to master and apply theoretical knowledge in this module. To the best of our knowledge, no previous study in veterinary education has specifically investigated the combination of a flipped classroom with mind mapping in the context of teaching ultrasound applications in animal husbandry.

## 2. Materials and Methods

### 2.1. Educational Design

The participants in this educational reform study were students from the Department of Veterinary Medicine, College of Animal Science and Veterinary Medicine, Xinyang Agriculture and Forestry University, who were third-year undergraduates enrolled in a four-year program (2023 cohort). The experimental group consisted of 20 female students and 10 male students; the control group consisted of 19 female students and 12 male students. None of these students had previously taken or failed the course. The course was worth 2.0 credits; the content focused on Chapter 11, Application of Ultrasound in Animal Husbandry (Syllabus refer to Supplementary Material). One class of 30 students was randomly selected as the experimental group by an independent researcher, who drew a class name from a container; another class of 31 students served as the control group. The sample size was determined by the actual enrollment in the two intact classes, not by an a priori power analysis. Owing to the nature of the teaching intervention, the students were not blinded to their group assignment. All the students were aware that they were participating in this study and that their personal information could not be identified. All quiz scores and questionnaire responses were anonymized prior to the analysis by removing any student identifiers. In accordance with the relevant institutional guidelines, formal ethical approval was waived for this educational intervention.

First, an overall framework for the course was established. This framework, which was developed following joint discussions among the course team, was divided into two parts: preclass preparation and practical classroom teaching (Table 1). Phase I (preclass preparation) lasted two weeks; Phase II (in-class teaching) was conducted over two class sessions (2 credit hours). Each group prepared one collective PowerPoint rather than individual presentations.

Before class, our teaching group conducted collective lesson planning and, on the basis of discussion, selected suitable units of content for flipped classroom teaching. In this study, ‘Ultrasound Applications in Animal Husbandry’ was designated as a knowledge unit for the teaching intervention. We subsequently developed various group teaching topics by reference to the curriculum (Table 2); students could select a topic to present on the basis of their own interests and form groups freely. All groups were tasked with preparing relevant teaching PowerPoints on the topics assigned to them. All the PowerPoint slides were reviewed by the instructor for assuring the accuracy of the content; no formal scoring rubric was applied.

While creating PowerPoint presentations, students could consult textbooks and reference books or use online resources, such as China National Knowledge Infrastructure (CNKI) (https://www.cnki.net/), the Super Star Learning APP, the Massive Open Online Course (MOOC) (https://www.icourse163.org/) and AI tools, to gather learning materials for the topic ‘Ultrasound Applications in Animal Husbandry’. Students were permitted to use AI tools to search for learning materials during their preclass preparation. Students could thus obtain an understanding of the background information pertaining to this chapter, as well as knowledge of veterinary imaging diagnostic and therapeutic techniques and advanced theories. The group members were required to have clearly defined roles, and each member was responsible for different teaching tasks, such as compiling materials, creating presentation slides or delivering classroom lectures. In addition, students were encouraged to incorporate case studies, animations and videos into their PowerPoint presentations to present otherwise dry veterinary imaging knowledge in an accessible and engaging manner, thereby successfully stimulating their interest in the subject.

The second stage involved practical classroom teaching, during which groups presented their findings. Each group selected a representative to deliver a PowerPoint presentation, outlining the key points that they had identified through their group work and sharing the challenges that they had encountered and the solutions that they had developed. When students subsequently posed questions, group members were required to discuss and respond. Where group members were unable to answer a particular question, teachers guided them to consult further resources and discuss the matter before providing an answer; the teacher also corrected any inaccuracies where necessary to ensure that all omissions were addressed. Each group was given approximately 5 min for the Q&A and discussion session. Ultimately, to consolidate the knowledge acquired by the students, the PowerPoint presentations that they submitted were collated and imported into the Doubao AI tool (Version 1.80.9, https://www.doubao.com) to generate a mind map. This tool was configured with temperature = 0.1 and top_*p* = 0.2, and the output was limited to three hierarchical levels with a 60% compression ratio; duplicate content was merged automatically. Parallel structural frameworks were applied to the pig and cattle/sheep sections, and the output was crosschecked by the instructor to standardize the terminology and eliminate mismatched information. During the revision process, teachers were primarily involved in this process to ensure the accuracy of key concepts, the logical correctness of the content, and the comprehensiveness of the presented knowledge. They made only minor adjustments, such as merging duplicate nodes or correcting erroneous branches. No formal scoring criteria were used during this revision process. The mind map was generated a few minutes after the PowerPoint file was uploaded.

### 2.2. Class Quiz

To assess the advantages of the combination of a flipped classroom with mind mapping over traditional classroom teaching, in-class tests were performed for students in the experimental group, who were subjected to the teaching reform, and for those in the control group, who received the traditional teacher-centered method. For the control group, the instructor delivered the same content via PowerPoint lectures; no pre-class preparation, group discussions, or student presentations were included. In-class tests were conducted via the Super Star Learning app in the form of online exams. The test consisted of 15 single-choice questions (30 points), 10 fill-in-the-blank questions (30 points), 10 true/false questions (30 points), and 1 short-answer question (10 points); its total duration was 90 min. Students were not permitted to review previous test papers or consult external materials. All the questions were completed independently by the students.

### 2.3. Questionnaire Survey

A questionnaire that covered aspects such as students’ interest in the course, level of knowledge acquisition, and experience of classroom interaction was designed. The purpose of this questionnaire was to effectively assess students’ subjective experiences and learning effectiveness under the ‘mind map & flipped classroom’ teaching model. The questionnaire was developed on the basis of the learning objectives of the flipped classroom and mind mapping intervention. No formal pilot testing or reliability analysis was conducted. Items A–E were categorical (i.e., multiple-choice with predefined options), whereas item F used a numerical rating scale ranging from 1 to 10. Descriptive statistics (percentages for items A–E; mean for item F) were used for the analysis. The survey results were collected using the WJX online tool (https://www.wjx.cn/). The questionnaire included the following questions:

A. Do you think mind maps assisted in understanding the key concepts covered in the ‘Veterinary Imaging’ course?
B. Do you think mind maps assisted in mastering the overall knowledge framework of ‘Veterinary Imaging’?
C. In the flipped classroom environment, how difficult do you find it to study the content of the ‘Veterinary Imaging’ course independently in advance?
D. In the classroom discussion session of the flipped classroom, how did you participate?
E. To what degree do you think the discussion sessions in the flipped classroom helped in learning ‘Veterinary Imaging’?
F. Please rate your satisfaction with the new teaching model (scale of 1–10).

### 2.4. Data Presentation

In this study, all the data collected were analyzed and graphed using GraphPad Prism (version 6.01) software. The normality of the score distributions was assessed by performing the Shapiro–Wilk test. Given that both groups exhibited deviations from normality (experimental group *p* = 0.032; control group *p* = 0.048), the Mann–Whitney U test was used to compare quiz scores between the two groups. The effect size was calculated as r = Z/√N. Only one primary comparison (concerning experimental and control group quiz scores) was performed; therefore, no adjustment for multiple comparisons was applied.

## 3. Results

### 3.1. Course Effectiveness and Mind Map Generation

**Figure 1:**
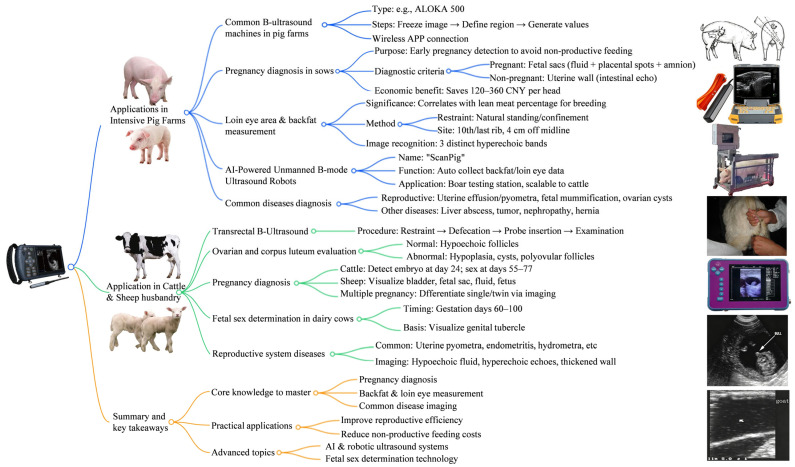
AI-generated mind maps of ultrasound applications in animal husbandry, organized from student PowerPoint content and generated by Doubao AI (https://www.doubao.com). The colors indicate different subtopics; the nodes represent key concepts extracted from the presentations. AI image generated with Doubao AI (https://www.doubao.com), current version as of 2025 (Doubao, Version 1.80.9), for educational illustration; input derived from presentations; no copyrighted material used.

Group members prepared PowerPoint presentations on their chosen topics, and on the basis of collaborative discussion, they approached difficult concepts within the topics, thereby achieving the learning objective of peer support. In this context, the students played dual roles as both designers and active participants in the learning activities. The practical teaching stages in the classroom were no longer teacher-centered. Instead, students engaged in a process of knowledge internalization through in-class presentations and group discussions, which greatly enhanced their active engagement in the learning process. Finally, the students’ presentation slides were collated and imported into an AI tool, resulting in the generation of a mind map (Figure 1).

### 3.2. Results of the Class Quiz

**Figure 2:**
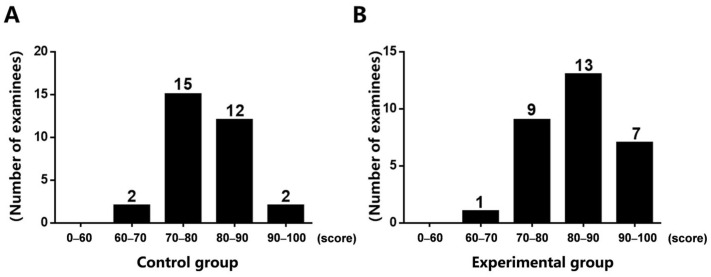
Distribution of student scores on a 100-point scale: (**A**) control group (n = 31); (**B**) experimental group (n = 30). Each bar represents the number of students contained within each 10-point score band.

As shown in the distribution chart of marks on a 100-point scale (Figure 2), the results of the students’ class tests indicated that both groups of students scored above 60, with no students failing. The Shapiro–Wilk test indicated that the score distributions in both groups deviated from normality (experimental group: *p* = 0.032; control group: *p* = 0.048). Notably, the experimental group had seven students who scored between 90 and 100, which was more than the two corresponding students in the control group; furthermore, the experimental group had thirteen students who scored between 80 and 90, accounting for the greatest number in any score band, whereas in the control group, the greatest number of students (15) was observed in the 70–80 score band. The results of the preliminary analysis revealed that compared with the control group, the experimental group had a greater proportion of high-achieving students, and the overall distribution of scores shifted toward the higher end of the scale. The mid-range (80–90 points) emerged as the core cluster, in sharp contrast with the control group’s distribution, which was concentrated in the 70–80 band. These findings reflect the fact that the students in the experimental group generally maintained positive attitudes toward learning and exhibited a high capacity for self-directed and proactive learning.

The Mann–Whitney U test revealed no statistically significant differences between the two groups (U = 361.0, z = −1.49, *p* = 0.135, r = 0.22). The mean difference was 3.79 points. A comparative statistical analysis revealed (Table 3) that the control class, which included 31 students, had an average score of 79.74, a standard deviation of 6.62, a maximum score of 90 and a minimum score of 67; the experimental class, which included 30 students, had an average score of 83.53, a standard deviation of 7.83, a maximum score of 98.0 and a minimum score of 69.0. The average score obtained by the students in the experimental class was 3.79 points higher than that obtained by the students in the control class. Furthermore, the standard deviation for the experimental class (7.83) was greater than that for the control class (6.62), suggesting that the distribution of scores was more dispersed in the experimental class. The score range for the experimental class was 29 points, whereas that for the control class was 23 points. In summary, the results of the experimental class exhibited considerably wider variation, whereas those of the control class were more concentrated. The highest score in the experimental class was 98, which was higher than that in the control class (90); however, the lowest score of 69 did not differ significantly from that in the control class (67), indicating that the internal variation within the experimental class was more pronounced. Although the difference did not reach statistical significance in this small cohort, the experimental group had higher mean scores and a greater number of top-scoring students (7 vs. 2 in the 90–100 range). The rightward shift in the experimental group’s score distribution (Figure 2) is consistent with this observed trend.

### 3.3. Questionnaire Results

To assess students’ learning impressions of the ‘flipped classroom combined with mind mapping’ teaching model in the ‘Veterinary Imaging’ course, we conducted a teaching-feedback inquiry, which ultimately resulted in the collection of 30 valid responses.

**Figure 3:**
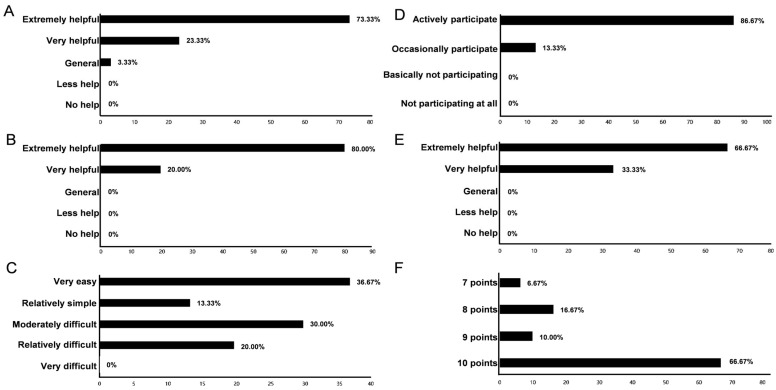
Student feedback on the ‘Mind Map and Flipped Classroom’ teaching model (n = 30): (**A**) Perceived help in the context of understanding key concepts within the course provided by mind maps; (**B**) Perceived help in the context of mastering the overall knowledge framework of the course provided by mind maps; (**C**) Perceived difficulty of independently prestudying veterinary ultrasonography content in the flipped classroom; (**D**) Students’ perceived level of participation in the flipped classroom discussion sessions; (**E**) Perceived help of flipped classroom discussions for learning veterinary imaging; (**F**) Students’ ratings of their levels of satisfaction with the new teaching model (1–10 scale). These six items were specifically designed for this study on the basis of the intervention’s learning objectives.

A detailed analysis of the questionnaire results revealed the following: As shown in Figure 3A, 73.33% of the students considered mind maps to be ‘extremely helpful’, 23.33% reported them as ‘quite helpful’, and only 3.33% rated them as ‘moderately helpful’ (no students reported that these maps were ‘somewhat unhelpful’ or ‘not helpful at all’). These findings indicate that mind maps are widely recognized as effective tools for facilitating the understanding of core course concepts. Eighty percent of the students regarded mind maps as ‘very helpful’, whereas 20% felt they were ‘somewhat helpful’; these findings indicate that mind maps were highly valued in terms of their ability to support students’ mastery of the overall knowledge framework for veterinary ultrasonography (Figure 3B). As shown in Figure 3C, 50% of the respondents considered selecting course content for independent study to be ‘very easy’ (36.67%) or ‘fairly easy’ (13.33%), whereas 30% described it as ‘moderately difficult’, with these two categories together accounting for 80% of the total. Only 20% of the students felt that this process was ‘fairly difficult’. As shown in Figure 3D, 86.67% of the students actively participated in discussions, whereas 13.33% participated occasionally. These findings indicate that the learning atmosphere in the flipped classroom was positive, as students were keen to share what they had learned with one another. As shown in Figure 3E, 66.67% of the students considered the discussion sessions in the flipped classroom to be extremely helpful for their studies in ‘Veterinary Imaging’, whereas 33.33% of the students considered those sessions to be quite helpful. None of the students rated these sessions as moderately helpful or lower, indicating that the discussion sessions were generally effective. Finally, the overall satisfaction scores for the new teaching model were collated, with the results indicating that 66.67% (20 students) were ‘very satisfied’, whereas 33.33% (10 students) gave scores of 7–9. No ratings ranging from ‘very dissatisfied’ to 6 were recorded, indicating a high level of overall satisfaction (Figure 3F).

Overall, our study revealed that the ‘mind mapping combined with the flipped classroom’ teaching model demonstrated positive results overall in the veterinary ultrasonography course. However, attention must also be given to students who struggle with preparatory work, those with low levels of participation in discussions, and those who face difficulties adapting to the new model.

## 4. Discussion

### 4.1. The Need for Innovation in Teaching in Veterinary Ultrasound Education

At present, many students are studying veterinary medicine, and faculty members face numerous pressing issues that require urgent attention. These issues include a relative shortage of teaching staff in veterinary ultrasound and the need to keep pace with rapid advancements in teaching methods and resources. Although various innovative tools, including ultrasound phantoms [15,16,17], 3D modeling and virtual reality simulations [18], and AI-powered platforms, have been developed to enhance training [19], integrating them into structured pedagogical frameworks remains challenging. If the aforementioned issues cannot be resolved in a timely manner, it will be difficult to guarantee the quality of teaching in this area. Ultrasound is not only a diagnostic and teaching tool in veterinary and medical education but also a clinically relevant therapeutic modality with measurable effects [20]. It is therefore necessary to actively explore new teaching models to encourage students to shift from passive to active learning and thereby enhance the quality of education for veterinary ultrasound professionals.

### 4.2. Overall Effectiveness of the Combination of a Flipped Classroom with Mind Mapping

With the increasing use of digital education, traditional classrooms are no longer able to meet the needs of students in the modern era, and a variety of teaching methods have emerged and achieved positive results in veterinary education [9,21,22], as well as in other health science disciplines, including nursing and medical education [23,24,25]. This study confirmed that the introduction of the flipped-classroom approach and mind mapping into the ultrasound module yielded positive results. Recent studies have reported similar benefits of flipped classroom approaches in ultrasound medical resident training [26] and digital intelligence-based teaching innovation in veterinary education [27]. Compared with traditional classroom teaching, this pedagogical reform significantly increased interactions between teachers and students, as well as peer support, while simultaneously increasing learning efficiency and student engagement. For instance, in the section on the application of ultrasound in intensive pig farming, the fourth subtopic focuses on the use of the AI-powered ultrasound robot ‘ScanPig’. An introduction to modern intelligent ultrasound equipment was needed, and students could familiarize themselves with new theories and technologies by consulting relevant academic literature and news reports. Each student in the group had a specific role, and during the group discussion, they could share what they had learned and their insights.

During our discussions with students, we observed that most of the learners felt that the classroom atmosphere was relaxed and that they had gained greater confidence, which is consistent with the findings of a previous veterinary education study [28].

Compared with other active learning methods (such as problem-based learning (PBL), case-based learning (CBL) and team-based learning (TBL)), which have been discussed in recent studies on veterinary and medical education [29,30,31], the combination of a flipped classroom with mind mapping considered in this study has several unique characteristics. PBL and CBL focus mainly on clinical reasoning through case scenarios, whereas our approach emphasizes independent preclass preparation and cooperative classroom knowledge construction. TBL allows a fixed group of students to participate in a group readiness assurance test, whereas our method facilitates flexible group formation and topic selection on the basis of students’ interests. Unlike those of other methods, the mind map generated by AI provides a visual framework for knowledge review. However, compared with these established methods, our method requires more self-regulation on the part of students; thus, it may not be suitable for all learners, as reflected by the increase in variance observed in the experimental group.

### 4.3. Differential Learning Outcomes and Challenges for Underperforming Students

However, in the process of teaching, we also observed that the knowledge points presented in the PowerPoint slides submitted by a very small number of groups were of mediocre quality, likely due to differences in students’ ability to collect learning materials and solve problems. Consequently, it is important to supervise preclass activities and implement appropriate measures to address any cases in which students lack sufficient preparation for a particular lesson, if necessary. To mitigate this issue, teachers provided guidance to students during the preparatory stage on an as-needed basis [32]. In fact, teachers primarily provided guidance on the basis of students’ needs rather than engaging in supervisory activities such as requiring students to sign in. Specifically, a lack of supervision may lead to inconsistencies in the quality of students’ preparation for class, which in turn may cause the true effectiveness of the teaching model to be underestimated and introduce bias. In the flipped classroom, knowledge acquisition no longer relies primarily on teacher-led instruction but is instead generated through peer-assisted learning and group discussions. This model may yield varying learning outcomes for students of different ability levels. An analysis of the in-class test results revealed that although the overall score distribution in the experimental group was biased toward the higher score range and that the experimental group’s highest score (98.0) and average score (83.53) were both higher than the control group’s highest score (90.0) and average score (79.74), the difference between the lowest score in the experimental group and that in the control group was not significant. Moreover, even with the adoption of a flipped-classroom approach combined with mind mapping, some students still did not benefit from it, which may be attributed to their low levels of engagement throughout the unit. The lack of benefit observed for low-performing students may be explained by their limited self-regulated learning skills and passive participation in group work. This situation thus widened the achievement gap, as evidenced by the larger standard deviation observed in the experimental group. Future instructional innovations should provide these students with more tutoring-based learning support.

### 4.4. The Role of Mind Mapping and the Integration of AI Tools

Mind mapping was first developed by the British scholar Tony Bazan in the field of educational psychology. The core goal of mind mapping is to construct a divergent node structure through the use of a variety of elements, such as words, lines, symbols and images, thereby transforming abstract, divergent thinking into an intuitive, visual tool [33]. Mind mapping converts complex textual information into a hierarchical diagram, thereby enhancing students’ ability to retain and retrieve information and improving their learning efficiency, as has been reported in the context of nursing education [34]. The introduction of mind maps enables students to consolidate fragmented knowledge points into a systematic network of knowledge, thereby both reinforcing immediate learning effectiveness and providing support for subsequent revisions. Previous research has indicated that the implementation of mind mapping and case-based learning in nursing education can effectively increase students’ theoretical knowledge, clinical skills and higher-order cognitive abilities while simultaneously resulting in higher levels of learning satisfaction [3]. In this study, we observed that conducting in-class exercises after all knowledge points were structured using mind maps significantly enhanced students’ learning effectiveness and fostered positive learning feedback. Notably, however, mind mapping can help students break down, assimilate and integrate knowledge. Studies in the field of dental education have also indicated that the implementation of mind mapping in the classroom requires structured guidance from teachers to enhance students’ cognitive structures and stimulate higher-order reasoning [35].

Currently, few studies have investigated the use of AI tools to generate mind maps for educational purposes. A study on language education revealed that students found mind maps generated by ChatGPT (free version) to be a highly useful teaching tool, as these maps could help breakdown complex ideas and make the relationships among concepts in a text visible [36]. In addition, the creation of mind maps using Monica AI was observed to significantly improve learning efficiency in healthcare education for people with psychosis, thereby helping them understand content more quickly so that they could apply what they learned to their daily lives [13]. In our study, AI tools were used to generate mind maps after the PowerPoint slides were collected. This approach not only significantly reduced the amount of time teachers spent creating diagrams but also made it easier for educators to provide structured guidance and targeted improvements on the basis of these diagrams. We therefore encourage teachers to incorporate this technology into their teaching practices.

### 4.5. Student Feedback, Challenges in Adaptation, and Future Improvements

The results of the questionnaire survey indicated that the novel teaching methodology received widespread approval among students. According to the survey results, the percentage of respondents who rated independent pre-class studies as ‘very easy’ (36.67%) or ‘fairly easy’ (13.33%) was 50%, whereas ‘moderately difficult’ ratings accounted for 30% of the total (these two categories jointly accounted for 80% of the total), and only 20% of the students considered this approach to be ‘fairly difficult’. These results reveal that the course should provide more basic preparatory guidance materials or a summary of key concepts for the 20% of students who reported that it was ‘quite difficult’. Notably, students who actively participate in class discussions are more likely to view previewing lessons as easy, whereas those who participate only occasionally or not at all tend to view that activity as difficult. In future lessons, we will encourage students to participate actively in discussions, as research has indicated that a positive attitude toward learning often leads to better academic results. Therefore, the task of stimulating students’ interest is vital [37,38]. In the questionnaire feedback section, a student commented that ‘this learning model lacks effective supervision and timely feedback’. Hence, we will establish a regular management system, including the introduction of weekly prestudy check-ins, online teacher consultation sessions, or peer-to-peer supervision within study groups, to ensure that students receive timely guidance during their independent study. Additionally, certain students struggled to adapt to the new teaching model. To address the difficulties experienced by these students in adapting to the new model, as well as the challenges faced by students with learning difficulties and those with moderate levels of satisfaction, we will implement training sessions on how to use the new model (such as tutorials on creating mind maps). Additionally, for students with learning difficulties, we provide personalized preparatory materials.

### 4.6. Limitations

This study was conducted at a single institution with a relatively small sample (N = 61) determined by class enrollment rather than an a priori power analysis. This fact limits the generalizability of the findings. Only short-term knowledge retention was assessed; long-term learning outcomes were not evaluated. Additionally, the questionnaire used in this study was developed ad hoc for this specific educational intervention and was not formally validated on the basis of pilot tests or reliability analyses. This fact limits the generalizability of the reported levels of satisfaction. In addition, the lack of individual-level randomization and the absence of blinding may have introduced confounding. The findings of this research may also not be generalizable to other courses or student populations. Given the small sample size, the findings of this study should be interpreted as preliminary. Furthermore, no pretests were conducted, the individual effects of the flipped classroom and mind mapping were not isolated, clinical skills were not directly measured, and AI-generated mind maps were not compared with student-generated mind maps.

## 5. Conclusions

This preliminary study highlights the educational potential of combining the flipped-classroom approach with mind mapping in the context of veterinary ultrasound education. This teaching practice was associated with improved classroom interaction and increased student engagement, as reflected in the questionnaire responses. It was also associated with a trend toward higher mean quiz scores and a greater proportion of high-scoring students, although this difference did not reach the level of statistical significance in this small cohort. However, students did not benefit equally from this approach. This study offers preliminary insights that may inform the development of innovative teaching models in the context of veterinary ultrasound education.

## Figures and Tables

**Table 1:** Teaching process for the experimental group, including the pre-class preparation and in-class teaching stages of the flipped classroom in combination with the mind mapping intervention.

Educational Stage	Detailed Teaching Steps
Preclass preparation	The teaching group determines the lesson topics in accordance with the syllabus.
	Topics were chosen on the basis of the students’ own interests, after which study groups were formed.
	Groups should search for study materials and create a PowerPoint presentation, after which the teacher reviews the presentation.
Practical teaching stage	Group representatives present PowerPoint presentations, while the other students pose questions.
	Group members discuss and answer the questions raised in the manner indicated above, and the teacher summarizes the issues with the goals of identifying and addressing any omissions.
	PowerPoint slides are collected and uploaded to an AI tool to generate a mind map; after revision, an in-class test is performed to assess students’ grasp of the content.

**Table 2:** Group presentation topics assigned to students in the experimental group, which are derived from the ‘Ultrasound Applications in Animal Husbandry’ module.

Subject Module	Topics for Group Presentations
The application of ultrasound in the context of intensive pig farming	Features and operational procedures used for B-ultrasound machines in pig farms.
	Ultrasound diagnosis methods for sow pregnancy.
	Procedure used to measure the area of the porcine eye muscle and backfat thickness.
	Principles and applications of AI-powered automated backfat measurement robots for pigs (ScanPig).
	Ultrasound diagnosis methods used for common diseases in pig farms.
The application of ultrasound in the context of cattle and sheep husbandry	Operational procedures used for transrectal B-mode ultrasonography in cattle and sheep.
	Ultrasound examination of follicles and corpus luteum in the ovaries of cattle and sheep.
	Ultrasound diagnostic methods for pregnant cows and ewes.
	Methods for ultrasound identification of calf sex.
	Ultrasound diagnostic methods used for reproductive diseases in cattle and sheep.

**Table 3:** Comparison of in-class quiz scores between the experimental group (n = 30) and control group (n = 31), including mean scores, standard deviations, and score ranges.

Group	Mean Score	Standard Deviation	Highest Score	Lowest Score
Experimental Group (n = 30)	83.53	7.89	98.0	69.0
Control Group (n = 31)	79.74	6.62	90.0	67.0

The Mann–Whitney U test revealed no statistically significant differences between the two groups (U = 361.0, z = −1.49, *p* = 0.135, r = 0.22). The mean difference was 3.79 points. Similar findings in veterinary education have been reported [14].

## Data Availability

All the data generated or analyzed during this study are included in this manuscript. The raw quiz scores and questionnaire responses are available from the corresponding author upon reasonable request.

## Supplementary Materials

The following supporting information can be downloaded at: https://www.mdpi.com/article/10.3390/ani16142129/s1. Syllabus for Veterinary Imaging. Refs. [39,40,41] are cited in the Supplementary Materials.
