# Supplementary material for: Preliminary Assessment of the Effect of a Flipped Classroom Combined with Mind Mapping on Learning Outcomes in Ultrasound Use in Animal Husbandry Education: A Comparison with Traditional Lecture-Based Learning Among Third-Year BS Students in China
Source: Animals (Basel). 2026 Jul 9;16(14):2129. doi: 10.3390/ani16142129 (PMC13404594; doi:10.3390/ani16142129)
Supplement: Supplementary file 1 [file animals-16-02129-s001.zip › animals-4375773-supplementary.pdf]

Syllabus for Veterinary Imaging

I. Basic Course Information

<table>
<tr><td>Course Name</td><td colspan="5">Veterinary Imaging</td></tr>
<tr><td>Course Type</td><td colspan="3">Major Courses</td><td>Credits</td><td>2.0</td></tr>
<tr><td>Total Hours</td><td>32</td><td>Lecture Hours</td><td>24</td><td>Practical Hours</td><td>8</td></tr>
<tr><td>Applicable Major</td><td colspan="5">Veterinary Medicine</td></tr>
<tr><td>Prerequisites</td><td colspan="5">Animal Anatomy, Animal Physiology, Animal Nutrition</td></tr>
</table>

II. Course Teaching Objectives

Specific Course Objectives

1. Master the basic principles and concepts of veterinary imaging, understand the talent demand in the industry, possess the basic qualities and skills required for practitioners, provide students with professional awareness cultivation and professional ethics education, improve students' comprehensive quality and professional competence. Enhance students' ability to adapt to career changes, lay the foundation for students' career development, and provide fundamental assurance for obtaining professional qualifications, employment, and entrepreneurial endeavors.

2. Systematically master the professional knowledge and theories of animal anatomy, animal physiology and biochemistry, and animal pathology; master the basic theoretical knowledge of imaging, understand the frontier knowledge and development trends of imaging. Master the basic knowledge and techniques of pet imaging examination; master the imaging characteristics of normal and diseased organs; proficiently interpret radiographs, sonograms, and electrocardiograms.

3. Through the integration of ideological and political elements into classroom teaching, enable students to have a firm and correct political direction, consciously establish and practice socialist core values, abide by laws and regulations, be realistic and pragmatic, possess good ideological and moral character, social ethics, professional ethics, and a strong sense of social responsibility; possess the value sentiment of knowing, loving, and serving agriculture.

III. Teaching Content

No.	Teaching Content	Teaching Methods	Hours	
			Lecture	Practical
1	Unit 1: Overview of Veterinary Imaging and X-ray Basics	Lecture Method	2	0
2	Unit 2: X-ray Imaging Techniques and Quality Assessment	Lecture Method	2	0
3	Unit 3: X-ray Examination of Canine and Feline Appendicular Skeleton and Joints	Lecture Method	2	2
4	Unit 4: X-ray Examination of Canine and Feline Respiratory System	Lecture Method	2	0
5	Unit 5: X-ray Examination of Canine and Feline Circulatory System	Lecture Method	2	0
6	Unit 6: X-ray Examination of Pet Abdomen	Lecture Method	2	0
7	Unit 7: Overview of Veterinary Ultrasound and Basic Principles	Lecture Method	2	2
8	Unit 8: Ultrasound Examination of the Caudal Abdomen in Pets	Lecture Method	2	2
9	Unit 9: Ultrasound Examination of the Mid-Abdomen in Pets	Lecture Method	2	0
10	Unit 10: Ultrasound Examination of the Hepatobiliary System in Pets	Lecture Method	2	0
11	Unit 11: Application of Ultrasound in Animal Husbandry	Flipped Classroom Combined with Mind Mapping	2	0
12	Unit 12: Electrocardiography in Pets	Lecture Method	2	2
**Total**			24	8

IV. Course Assessment

Specific evaluation methods may include: course questionnaires, interviews, course assessment score analysis, student evaluation of teaching, teacher evaluation of learning, teaching supervision, etc. Identify shortcomings through evaluation for subsequent continuous improvement.

V. Course Resource Library

(1) Recommended Textbooks:

[1] Xie F.Q. Veterinary Imaging. Beijing: China Agricultural University Press, 2019.

(2) Reference Books:

[1] Zhang H.B. Small Animal Clinical Diagnostics. Beijing: China Agricultural University Press, 2016.

[2] Deng G.Z. Veterinary Clinical Diagnostics. Beijing: China Agricultural University Press, 2009.

(3) Recommended Websites:

China Veterinary Practitioner Network: http://www.cnsav.com/

Chinese University MOOC: https://www.icourse163.org/

Smart Vocational Education: https://www.icve.com.cn/

This syllabus is formulated in accordance with the 2021 Undergraduate Talent Training Program.
